# Public hospitalizations for stroke in Brazil from 2009 to 2016

**DOI:** 10.1371/journal.pone.0213837

**Published:** 2019-03-19

**Authors:** Leila F. Dantas, Janaina F. Marchesi, Igor T. Peres, Silvio Hamacher, Fernando A. Bozza, Ricardo A. Quintano Neira

**Affiliations:** 1 Department of Industrial Engineering, Pontifical Catholic University of Rio de Janeiro, Rio de Janeiro, RJ, Brazil; 2 Evandro Chagas National Institute of Infectious Disease, Oswaldo Cruz Foundation (FIOCRUZ), Rio de Janeiro, RJ, Brazil; 3 IDOR, D’Or Institute for Research and Education, Rio de Janeiro, RJ, Brazil; Universidade do Extremo Sul Catarinense, BRAZIL

## Abstract

**Background:**

Stroke is the third major cause of death in the world and the second in Brazil. The purpose of this work was to assess the stroke-related hospitalization, in-hospital mortality, and case fatality rates under the Brazilian Unified Health System (SUS) from 2009 to 2016.

**Methods:**

We evaluated the hospital admissions for stroke and their associated outcomes using data from the Hospital Information available at the Informatics Department of SUS. We selected hospitalization registries according to stroke diagnosis codes from the International Statistical Classification of Diseases and Related Health Problems (ICD-10). We identified the association of age and sex with patient death through multiple logistic regression and calculated the rates of hospitalization, mortality and case-fatality per 100,000 inhabitants using age-adjustment methodology.

**Results:**

We analyzed 1,113,599 stroke hospitalizations. From 2009 to 2016, the number of admissions increased from 131,122 to 146,950 and the absolute number of in-hospital deaths increased from 28,731 to 31,937. Younger age and male sex were significantly associated with patient survival. Our results showed that the annual age-adjusted hospitalization and in-hospital mortality rates decreased by 11.8% and 12.6%, respectively, but the case fatality rate increased for patients older than 70 years.

**Conclusions:**

Although the age-adjusted hospitalization and in-hospital mortality rates declined, the total number of hospitalization and deaths have increased. It is expected a continuous increase over the next years of stroke admissions with the rapid aging of the Brazilian population. Efforts should be renewed targeting risk factors, access to hospital and rehabilitation in particular for the elderly population.

## Introduction

Stroke is the third leading cause of death in the world [1], resulting in 5.9 million deaths and 49.9 million cases in 2010 [2]. In Brazil, stroke was responsible for 10.18% of all deaths in the country in 2009 [3] and the fourth leading cause of years of life lost in 2016 [4,5]. The risk of premature death due to this disease is one of the highest in the world [6].

Despite a decline in mortality in the last decades and advances in diagnosis and treatment [3], stroke still represents a major challenge and continues to impose a significant burden on the Brazilian Unified Health System (*Sistema Único de Saúde*–SUS). SUS is one of the largest national (universal) health systems in the world [7], but there are still shortages of public funding, resource misallocation and other weaknesses in its services [8]. Understanding changes in admission and death related to stroke over time are essential to the development of better public health policies, resource allocation, and case management recommendations [9,10].

Studies on stroke in Brazil were recently published. Marinho et al. [4] presented an analysis of health patterns and risk factors among the Brazilian states, showing trends in mortality rates and years of lost life caused by cerebrovascular disease. Santana et al. [11] presented an epidemiological report of stroke considering the whole country. Stroke mortality in specific Brazilian cities was discussed in different research studies [12,13,14]. Goulart et al. [15] and Minelli at al. [16] analyzed the stroke incidence and case fatality in specific Brazilian cities. Adami et al. [17] investigated hospitalizations for stroke in Brazil considering a restrict age group (15–49 years old) from 2008 to 2012. Therefore, updated information on hospital admissions, in-hospital mortality and case fatality for stroke considering the whole country and broad age range is still scarce in Brazil.

Given the concerns related to stroke assistance, this study reviewed eight years of stroke public admissions and in-hospital mortality in Brazil. The aims of this study were: (1) to describe the hospital admissions and deaths for stroke under SUS; (2) to assess stroke-related hospitalization, in-hospital mortality, and case fatality rates per 100,000 inhabitants for each year, age group and sex, using age-adjustment methodology.

## Materials and methods

### Data collection

We extracted data from two databases available for public access. The first dataset, SIHSUS, was obtained from SUS, which is available at the DATASUS website [18]. DATASUS is the Informatics Department of the Brazilian Unified Health System, Ministry of Health, Brazil. The SIHSUS presents the Authorizations for Hospitalization (AIH–*Autorização de Internação Hospitalar*). Each AIH registry contains de-identified data on inpatient demographic characteristics, length of stay, patient outcome (i.e., discharge to home or death) and other information (the details on the database attributes used in this work can be found in S1 Table). The second one, the population dataset, was obtained from the Brazilian Institute of Geography and Statistics (IBGE—*Instituto Brasileiro de Geografia e Estatística*) [19] for each year, age and sex. We included this information in the S2 Table.

We performed the analysis using secondary data containing no information that could identify patients. All data were anonymized and aggregated before access and analysis. Therefore, the ethics principles were respected and informed consent is not required.

### Study population

The Brazilian Unified Health System allows all Brazilian citizens and permanent residents to have access to hospital and clinical care. The population projection of Brazil in 2016 was 206,081,432 compared to 193,543,969 in 2009 [19]. Of these, more than three-fourths depend exclusively on the SUS for health services [7].

The AIH database registered 89,135,303 hospital admissions from January 1^st^, 2009 to December 31^st^, 2016. We included in the analysis only hospitalizations with patient outcomes of “discharge to home” or “death”, aiming to analyze the treatment effectiveness (patient lives or dies). Hospitalization registries with patients younger than 20 years old and with the length of stay (LOS) greater than 60 days were excluded. Therefore, the total number of hospital admissions was 57,102,743.

From this subset of hospitalizations, we selected cases that had the primary diagnosis of stroke, considering all diagnosis codes from the International Statistical Classification of Diseases and Related Health Problems (ICD-10) [20] categories I60, I61, I63 and I64 as was done in previous studies [10, 17]. These codes include subarachnoid hemorrhage, intracerebral hemorrhage (ICH), ischemic stroke, and acute stroke of undetermined cause.

### Data analyses

Considering the inclusion criteria above, annual crude rates of hospital admissions for stroke and the outcome of these hospitalizations were calculated according to the sex and age groups. After that, using the population data, we calculated the hospitalization rate, in-hospital mortality rate and case fatality rate per 100,000 inhabitants.

Case fatality is the proportion of patients who died from any cause during their admission with a diagnosis of stroke. Specifically, the numerator is the in-hospital mortality rate and the denominator the hospitalization rate.

Aiming to remove the effects regarding the diversity of the population age structure, we used the age-adjustment methodology to calculate new total rates. For each year, age-standardized estimates for total hospitalization, in-hospital mortality and case fatality rates were calculated, using the IBGE Brazilian population [19] and the World Health Organization (WHO) world population as the reference [21]. This same methodology was used in other previous stroke studies [2,16,17,22] and it is detailed in the S1 Appendix. The S3 Table presents the formula and description of each of the indicators used in this study.

In addition, we evaluated the association of independent variables (age and sex) with the patient’s death through a multiple logistic regression model [23]. This analysis determines if these factors influence the case fatality rate. A factor was statistically significant when the p-value was less than 0.05. We randomly divided the hospitalization registries into training and test sets (containing 80% and 20% of the data, respectively). All statistical analyses were done using R software version 3.4.3. [24].

## Results

From 57,102,743 hospital admissions included in the study, 1,113,599 (1.9%) were admissions with a primary diagnosis associated with stroke codes. Table 1 shows the number of stroke admissions and in-hospital deaths in SUS over the 8 years for each age group and sex. We observed that the absolute number of stroke admissions increased by 12.1%, from 131,122 in 2009 to 146,950 in 2016. The absolute number of in-hospital deaths increased by 11.2%, from 28,731 to 31,937 deaths during the study period. The proportion of in-hospital deaths due to stroke over all in-hospital deaths from 2009 to 2016 was 7.4%.

**Table 1:** Annual crude rates for hospital admissions with primary diagnosis of stroke and the number of in-hospital deaths from these hospitalizations according to the sex and age group from 2009 to 2016.

**Stroke admissions**	**2009**	**2010**	**2011**	**2012**	**2013**	**2014**	**2015**		**2016**	**Δ%**		**Annual Average (SD)**
**Total**	131,122	135,742	138,794	137,275	139,075	141,129	143,512		146,950	12.1%		138,935 (4,832)
**Age group**												
20 to 29 years	1,945	2,096	1,992	1,934	1,883	1,884	1,895		1,929	-0.8%		1,945 (71)
30 to 39 years	4,512	4,492	4,586	4,524	4,417	4,585	4,649		4,765	5.6%		4,566 (107)
40 to 49 years	12,206	11,996	12,324	11,663	11,597	11,677	11,760		11,895	-2.5%		11,890 (267)
50 to 59 years	22,496	22,847	23,154	22,928	23,056	23,323	23,678		24,022	6.8%		23,188 (483)
60 to 69 years	30,332	31,196	31,925	32,459	33,094	33,936	34,873		36,221	19.4%		33,005 (1,949)
70 to 79 years	34,310	35,545	36,672	35,565	36,037	36,489	37,089		37,583	9.5%		36,161 (1,030)
More than 80 years	25,321	27,570	28,141	28,202	28,991	29,235	29,568		30,535	20.6%		28,445 (1,569)
**Sex **												
Male	67,001	69,556	71,091	70,283	71,036	72,632	73,999		75,885	13.3%		71,435 (2,741)
Female	64,121	66,186	67,703	66,992	68,039	68,497	69,513		71,065	10.8%		67,765 (2,102)
**In-hospital deaths for stroke**	**2009**	**2010**	**2011**	**2012**	**2013**	**2014**	**2015**		**2016**	**Δ%**		**Annual Average (SD)**
**Total**	28,731	29,958	29,591	28,858	28,859	29,272	30,728		31,937	11.2%		29,432 (1,112)
**Age group **												
20 to 29 years	405	423	314	279	287	290	266		331	-18.3%		324 (59)
30 to 39 years	988	996	864	771	742	768	870		871	-11.8%		859 (97)
40 to 49 years	2,686	2,643	2,486	2,208	2,236	2,134	2,221		2,228	-17.1%		2,355 (217)
50 to 59 years	4,632	4,494	4,256	4,158	4,068	4,214	4,443		4,437	-4.2%		4,338 (192)
60 to 69 years	5,881	6,078	6,065	6,059	6,067	6,173	6,544		6,903	17.4%		6,221 (334)
70 to 79 years	7,347	7,728	7,675	7,650	7,429	7,610	7,936		8,390	14.2%		7,721 (325)
More than 80 years	6,792	7,596	7,931	7,733	8,030	8,083	8,448		8,777	29.2%		7,924 (593)
**Sex**												
Male	14,464	15,126	14,636	14,373	14,413	14,635	15,273		15,887	9.8%		14,851 (533)
Female	14,267	14,832	14,955	14,485	14,446	14,637	15,455		16,050	12.5%		14,891 (596)

SD, standard deviation.

The group of patients with age between 70 and 79 years old had the highest annual average of admissions, representing 26% of the total, followed by the group with age between 60 and 69 years old (23.7%) and patients with more than 80 years old (20.4%). Very old patients had the highest average case fatality rate (27.8%), while the overall annual average was at 21.4%.

**Fig 1:**
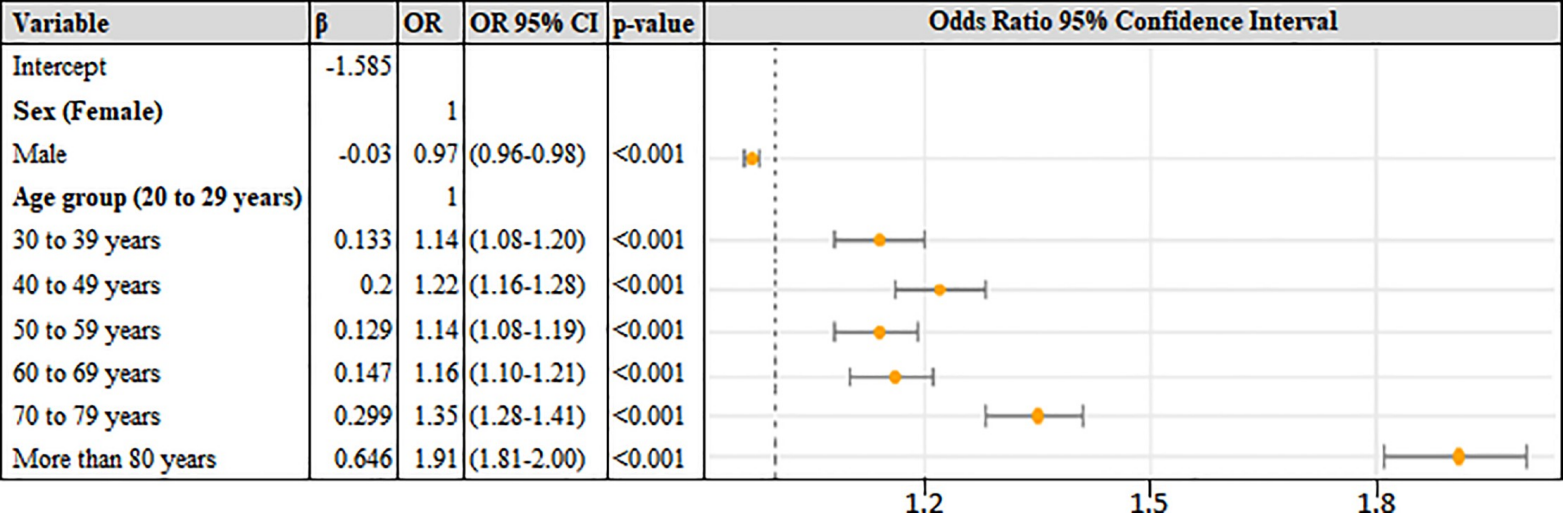
Results of multiple logistic regression analysis of factors associated with fatality rate. The coefficients, Odds Ratio (ORs), ORs with 95% Confidence Interval (CI) and p-values for each independent variable are represented. The reference level for each category is indicated in parentheses. The ORs 95% CI can be better visualized on the right side.

From the multiple logistic regression analysis, we found that age (p < 0.001) and sex (p < 0.001) were significantly associated with the case fatality rate. Fig 1 presents the results of the statistical analysis.

Analyzing Fig 1, we can note that very old patients were 1.91 times more likely to die than the group of patients with age between 20 and 29 years old. Although the difference is slight, men (OR = 0.97) are more likely to survive than women. The accuracy of the developed model was 79%.

Table 2 shows the age-adjusted rates for each sex and each age group. The annual age-adjusted hospitalization rate decreased by 11.8% (from 120.1 in 2009 to 105.9 in 2016), and the annual age-adjusted in-hospital mortality rate decreased by 12.6% (from 26.3 to 23.0). The age-adjusted case fatality rate kept almost constant during the study period, with an annual average of 21.4%.

**Table 2:** Age-adjusted hospitalization rate, age-adjusted in-hospital mortality rate and case-fatality rate per 100,000 inhabitants for each age group and sex from 2009 to 2016.

Age-adjusted hospitalization rate^1^	2009	2010	2011	2012	2013	2014	2015	2016	Δ%
**Total**	**120.1**	**120.2**	**118.8**	**113.5**	**111.2**	**109.0**	**107.1**	**105.9**	**-11.8%**
**Age group**									
20 to 29 years	5.5	6.0	5.7	5.5	5.4	5.5	5.5	5.7	2.1%
30 to 39 years	15.3	14.9	14.9	14.3	13.7	13.9	13.9	14.1	-8.0%
40 to 49 years	49.4	47.9	48.5	45.3	44.6	44.3	44.0	43.8	-11.5%
50 to 59 years	126.2	123.9	121.4	116.3	113.4	111.4	110.2	109.1	-13.5%
60 to 69 years	288.1	284.4	278.8	271.2	264.5	259.6	255.6	254.8	-11.5%
70 to 79 years	594.0	597.0	597.6	562.3	552.4	541.1	530.6	517.6	-12.9%
More than 80 years	989.0	1,029.5	1,006.5	967.3	953.9	922.3	893.6	883.0	-10.7%
**Sex**									
Male	109.1	111.3	111.7	108.5	107.9	108.5	108.8	109.8	0.6%
Female	99.2	100.5	100.9	98.1	97.9	96.9	96.7	97.3	-1.9%
**Age-adjusted in-hospital mortality rate**^1^	**2009**	**2010**	**2011**	**2012**	**2013**	**2014**	**2015**	**2016**	**Δ%**
**Total**	**26.3**	**26.5**	**25.3**	**23.9**	**23.1**	**22.6**	**22.9**	**23.0**	**-12.6%**
**Age group**									
20 to 29 years	1.2	1.2	0.9	0.8	0.8	0.8	0.8	1.0	-15.9%
30 to 39 years	3.3	3.3	2.8	2.4	2.3	2.3	2.6	2.6	-23.2%
40 to 49 years	10.9	10.5	9.8	8.6	8.6	8.1	8.3	8.2	-24.7%
50 to 59 years	26.0	24.4	22.3	21.1	20.0	20.1	20.7	20.2	-22.4%
60 to 69 years	55.9	55.4	53.0	50.6	48.5	47.2	48.0	48.6	-13.1%
70 to 79 years	127.2	129.8	125.1	121.0	113.9	112.8	113.5	115.5	-9.2%
More than 80 years	265.3	283.7	283.7	265.2	264.2	255.0	255.3	253.8	-4.3%
**Sex**									
Male	23.6	24.2	23.0	22.2	21.9	21.9	22.5	23.0	-2.4%
Female	22.1	22.5	22.3	21.2	20.8	20.7	21.5	22.0	-0.4%
**Age-adjusted case fatality rate**	**2009**	**2010**	**2011**	**2012**	**2013**	**2014**	**2015**	**2016**	**Δ%**
**Total**	**21.9%**	**22.1%**	**21.3%**	**21.1%**	**20.8%**	**20.7%**	**21.4%**	**21.7%**	**-0.9%**
**Age Group**									
20 to 29 years	20.8%	20.2%	15.8%	14.4%	15.2%	15.4%	14.0%	17.2%	-17.6%
30 to 39 years	21.9%	22.2%	18.8%	17.0%	16.8%	16.8%	18.7%	18.3%	-16.5%
40 to 49 years	22.0%	22.0%	20.2%	18.9%	19.3%	18.3%	18.9%	18.7%	-14.9%
50 to 59 years	20.6%	19.7%	18.4%	18.1%	17.6%	18.1%	18.8%	18.5%	-10.3%
60 to 69 years	19.4%	19.5%	19.0%	18.7%	18.3%	18.2%	18.8%	19.1%	-1.7%
70 to 79 years	21.4%	21.7%	20.9%	21.5%	20.6%	20.9%	21.4%	22.3%	4.3%
More than 80 years	26.8%	27.6%	28.2%	27.4%	27.7%	27.6%	28.6%	28.7%	7.2%
**Sex**									
Male	21.6%	21.7%	20.6%	20.5%	20.3%	20.1%	20.6%	20.9%	-3.0%
Female	22.3%	22.4%	22.1%	21.6%	21.2%	21.4%	22.2%	22.6%	1.5%

^1^ rates per 100,000 inhabitants

From Table 2, we can observe that the age-adjusted hospitalization rate decreased for all age groups from 2009 to 2016, except for younger patients, in which there was an increase of 2.1% of admissions. Regarding the in-hospital mortality rate, there was a reduction for all age groups. In terms of case fatality rate, we can observe an increase for patients older than 70 years.

We can note that men had higher hospitalization and in-hospital mortality rates (on average 109.5 and 22.8 respectively) than women (on average 98.44 and 21.64 respectively) during all study period. However, the case fatality was higher for women (21.9%) than men (20.8%).

## Discussion

The national data of public hospitalizations for stroke in Brazil from 2009 to 2016 showed that 1.9% of all admissions were associated with stroke diagnostic (ICD-10 codes from categories I60, I61, I63, and I64). The absolute number of stroke admissions and in-hospital deaths increased by 12.1% and 11.2%, respectively. This phenomenon may be partially explained by the population growth [25], which increased by 8.2% during the same period [19], the rapid aging of the Brazilian population and the increase in life expectancy [9,25,26].

During the study period, the Brazilian population growth was higher for very old patients (35.1%), followed by the age groups with 60–69 (35.0%), 70–79 (25.7%) and 50–59 years old (23.5%). According to IBGE projections [19], people older than 50 years will correspond to 31.2% of all Brazilian population in 2030 and 47.6% in 2060. During these 30 years, the proportion of very old (more than 80 years) will increase, approximately, by 193%. We observed that the higher the age, the greater the hospitalization and in-hospital mortality rates. Therefore, it is important for the healthcare system to be prepared for the future, as the number of stroke cases is expected to increase due to population aging.

Regarding the age-adjusted data, we can observe that the age-adjusted hospitalization rate decreased by 11.8% in the period analyzed, and this drop was found for all age groups, except for the group from 20–29 years old. This reduction may be explained by the improvement in the control of the main risk factors as well as greater population concern with health care and prevention. According to O’Donnell et al., approximately 90% of the population-attributable risk of stroke is associated with ten potentially modifiable risk factors: hypertension, smoking, diabetes mellitus, physical activity, diet, psychosocial factors, abdominal obesity, alcohol, cardiac causes, and apolipoproteins [27].

The prevalence of smokers in Brazil decreased by 0.62% on average per year, from 2006 to 2013 for both sexes [28]. For hypertension public hospitalizations, data indicates a significant reduction of 54.9% from 2000 to 2013 [29], and the meta-analysis by Picon et al. showed a reducing trend in the prevalence of hypertension in the country in the last three decades [30]. However, other studies pointed to an increase in the prevalence of obesity and diabetes, showing that overweight patients increased by 26.3% and obesity increased by 60% from 2006 to 2016 [31]. The crude prevalence of diabetes has trended upward over the years, increasing 0.26% per year from 2006 to 2014 [32]. Therefore, we can note a reduction in the prevalence of smokers and in hospitalizations for hypertension (under SUS), which might justify the decrease in the stroke hospitalization rate for the majority of age groups, even with a tendency of worsening rates of metabolic diseases.

Besides better attention for related risk factors, other efforts could have contributed to the decline in stroke admissions, including improved access to primary care and the potential impact of new technologies to detect and treat non-communicable diseases [4].

The age-adjusted in-hospital mortality rate decreased by 12.6% in this 8-year period. This in-hospital mortality reduction was observed in all ages and genders, being more significant for younger patients than for older ones. According to Lackland et al., the implementation of interventions and programs to reduce stroke risk also can contribute to a reduction in mortality [33]. However, improving in-hospital mortality rates may imply the need for extra supportive services, since more patients may require rehabilitation or long-term care services as more stroke patients survive [34].

The multiple logistic regression analysis concluded that age group is a significant variable to explain the case fatality: the older the patient, the greater the likelihood they would die when diagnosed with stroke. According to Santana et al., the stroke affected more Brazilian men and individuals over the age of 70 in 2016 [11]. The case fatality rate kept constant during the study period with an annual average of 21.4%, showing a reduction for almost all age groups. However, we observed an increase in the case fatality rate for age groups of patients more than 70 years old. This increase is worrisome since the number of stroke admissions is expected to increase for these age groups due to the aging population.

Men had higher hospitalization and in-hospital mortality rates than women, but the opposite was seen for case fatality rate. This last finding can be explained by the fact that the proportion of older women was greater than older men and older patients were more likely to die. Unlike other works [2,35], the reduction in stroke case fatality rates was observed only for the male sex.

This study has limitations. Only public hospitalization data were analyzed, so it excludes stroke patients who were not admitted to SUS (mostly patients with private insurance). Besides that, we worked with administrative data and there exists the possibility of coding errors. We were not able to verify diagnoses or stroke severity since clinical information was not available in the data and the analyses by stroke subtype were also not possible because of the high proportion of unspecified strokes (I64 code represented on average 77% of all stroke cases). Finally, since each patient did not have an identification code, it was not possible to identify whether the admitted patient was a new case of stroke or a return patient. In the future, we aim to analyze the association among availability of resources, stroke hospitalization and mortality rates, looking at regional trends and identifying strategies for vulnerable populations.

## Conclusions

This study reviewed eight years of public hospital admissions for stroke and their outcomes in Brazil. Although the age-adjusted hospitalization and in-hospital mortality rates declined by 11.8% and 12.6% in the period analyzed, respectively, the increase case-fatality rate was pronounced among older individuals. This increase is worrisome since the stroke admissions are expected to increase over the next years for these age groups due to population aging. Therefore, policies and efforts should be renewed targeting risk factor control and improving access and care to vulnerable people in the Brazilian Public System.

## Supporting information

S1 Table List of attributes from SIHSUS database used in this work. (PDF)

S2 Table Population projection data obtained from the Brazilian Institute of Geography and Statistics for each year, age and sex. (PDF)

S1 Appendix Age-adjustment methodology. (PDF)

S3 Table Formulation and description of the indicators used during the study. (PDF)

## References

[1] Thrift AG, Cadilhac DA, Thayabaranathan T, Howard G, Howard VJ, Rothwell PM, et al Global stroke statistics. Int J Stroke. Wiley Online Library; 2014;9: 6–18. 10.1111/ijs.12245 24350870

[2] Feigin VL, Forouzanfar MH, Krishnamurthi R, Mensah GA, Connor M, Bennett DA, et al Global and regional burden of stroke during 1990–2010: findings from the Global Burden of Disease Study 2010. Lancet. Elsevier; 2014;383: 245–255. 24449944 10.1016/s0140-6736(13)61953-4 PMC4181600

[3] Garritano CR, Luz PM, Pires MLE, Barbosa MTS, Batista KM. Analysis of the mortality trend due to cerebrovascular accident in Brazil in the XXI century. Arq Bras Cardiol. SciELO Brasil; 2012;98: 519–527. 22534777 10.1590/s0066-782x2012005000041

[4] Marinho Fatima, et al "Burden of disease in Brazil, 1990–2016: a systematic subnational analysis for the Global Burden of Disease Study 2016." The Lancet 392 10149 (2018): 760–775. 10.1016/S0140-6736(18)31221-2 PMC6123514 30037735

[5] GBD 2016 Lifetime Risk of Stroke Collaborators. "Global, Regional, and Country-Specific Lifetime Risks of Stroke, 1990 and 2016." New England Journal of Medicine 379 25 (2018): 2429–2437. 10.1056/NEJMoa1804492 30575491 PMC6247346

[6] Lotufo PA. Stroke is still a neglected disease in Brazil. Sao Paulo Med J. SciELO Brasil; 2015;133: 457–459. 10.1590/1516-3180.2015.13360510 26760122 PMC10496556

[7] CSIS. Brazil's Sistema Único da Saúde (SUS): Caught in the Cross Fire [Internet]. [cited 05 Feb 2019]. Available:https://www.csis.org/blogs/smart-global-health/brazils-sistema-unico-da-saude-sus-caught-cross-fire

[8] Massuda Adriano, et al "The Brazilian health system at crossroads: progress, crisis and resilience." BMJ global health 3 4 (2018): e000829 10.1136/bmjgh-2018-000829 29997906 PMC6035510

[9] Béjot Y, Bailly H, Durier J, Giroud M. Epidemiology of stroke in Europe and trends for the 21st century. Presse Med. Elsevier; 2016;45: e391—e398. 10.1016/j.lpm.2016.10.003 27816343

[10] Sarfo FS, Akassi J, Awuah D, Adamu S, Nkyi C, Owolabi M, et al Trends in stroke admission and mortality rates from 1983 to 2013 in central Ghana. J Neurol Sci. Elsevier; 2015;357: 240–245. 10.1016/j.jns.2015.07.043 26293417

[11] de Santana Nathalia Matos, et al "The burden of stroke in Brazil in 2016: an analysis of the Global Burden of Disease study findings." BMC research notes 11 1 (2018): 735 10.1186/s13104-018-3842-3 30326942 PMC6192154

[12] de Carvalho JJF, Alves MB, Viana GÁA, Machado CB, dos Santos BFC, Kanamura AH, et al Stroke Epidemiology, Patterns of Management, and Outcomes in Fortaleza, Brazil. Stroke. Am Heart Assoc; 2011;42: 3341–3346. 10.1161/STROKEAHA.111.626523 22052521

[13] Lotufo PA, Goulart AC, Bensenor IM. Race, gender and stroke subtypes mortality in São Paulo, Brazil. Arq Neuropsiquiatr 2007;65(3-B):752–757. 17952275 10.1590/s0004-282x2007000500004

[14] Cabral NL, Gonçalves ARR, Longo AL, Moro CHC, Costa G, et al Trends in stroke incidence, mortality and case fatality rates in Joinville, Brazil: 1995–2006. J Neurol Neurosurg Psychiatry 2009;80:749–754. 10.1136/jnnp.2008.164475 19147630 PMC2688773

[15] Goulart Alessandra C., et al "A stepwise approach to stroke surveillance in Brazil: the EMMA (Estudo de Mortalidade e Morbidade do Acidente Vascular Cerebral) study." International Journal of Stroke 5 4 (2010): 284–289. 10.1111/j.1747-4949.2010.00441.x 20636711

[16] Minelli C, Fen LF, Minelli DPC. Stroke incidence, prognosis, 30-day, and 1-year case fatality rates in Matao, Brazil. Stroke. Am Heart Assoc; 2007;38: 2906–2911. 10.1161/STROKEAHA.107.484139 17916767

[17] Adami F, Figueiredo FWS, Paiva LS, Sá TH, Santos EFS, Martins BL, et al Mortality and incidence of hospital admissions for stroke among Brazilians aged 15 to 49 years between 2008 and 2012. PLoS One. Public Library of Science; 2016;11: e0152739 10.1371/journal.pone.0152739 27332892 PMC4917086

[18] DATASUS. Departamento de Informática do SUS [Internet]. [cited 17 Apr 2017]. Available: http://datasus.saude.gov.br/

[19] IBGE. Instituto Brasileiro de Geografia e Estatística (Brazilian Institute of Geography and Statistics) [Internet]. [cited 17 Apr 2017]. Available: http://www.ibge.gov.br/home/

[20] WHO. International classification of diseases, 10th revision. Geneva: http://www.who.int/en/; 2007.

[21] Ahmad OB, Boschi-Pinto C, Lopez AD, Murray CJ, Lozano R, Inoue M. Age Standardization of rates: A new who standard. In: Organization WH, editor. 2001. p. 1–14.

[22] Passos V, Ishitani LH, Franco GC, Lana GC, Abreu DMX, Marinho M de F, et al Consistent declining trends in stroke mortality in Brazil: mission accomplished? Arq Neuropsiquiatr. SciELO Brasil; 2016;74: 376–381. 10.1590/0004-282X20160055 27191233

[23] Rolim CLRC, Martins M. Quality of care for ischemic stroke in the Brazilian Unified National Health System. Cad Saude Publica. SciELO Public Health; 2011;27: 2106–2116. 22124488 10.1590/s0102-311x2011001100004

[24] Team RStudio. Integrated Development for R. RStudio, Inc., Boston, MA: RStudio; 2015.

[25] Hankey GJ. No Title. Lancet. 2016;389: 641–654. 10.1016/S0140-6736(16)30962-X 27637676

[26] Béjot Y, Daubail B, Giroud M. Epidemiology of stroke and transient ischemic attacks: Current knowledge and perspectives. Rev Neurol (Paris). Elsevier; 2016;172: 59–68. 26718592 10.1016/j.neurol.2015.07.013

[27] O’Donnell MJ, Chin SL, Rangarajan S, Xavier D, Liu L, Zhang H, et al Global and regional effects of potentially modifiable risk factors associated with acute stroke in 32 countries (INTERSTROKE): a case-control study. Lancet. Elsevier; 2016;388: 761–775. 10.1016/S0140-6736(16)30506-2 27431356

[28] Malta DC, Oliveira TP, Luz M, Stopa SR, Silva Junior JB, Reis AAC. Tendências de indicadores de tabagismo nas capitais brasileiras, 2006 a 2013. Ciênc Saúde Coletiva. SciELO Public Health; 2015;20: 631–640.

[29] Malachias MVB, Souza WKSB, Plavnik FL, Rodrigues CIS, Brandão AA, Neves MFT et al. 7a Diretriz Brasileira de Hipertensão Arterial. Rio de Janeiro; 2016.

[30] Picon R V, Fuchs FD, Moreira LB, Riegel G, Fuchs SC. Trends in prevalence of hypertension in Brazil: a systematic review with meta-analysis. PLoS One. Public Library of Science; 2012;7: e48255 10.1371/journal.pone.0048255 23118964 PMC3485225

[31] Monteiro CA, Maia EG, Machado IE, Nico LS, Santos MAS, de Souza M. Vigitel Brasil 2006: vigilância de fatores de risco e proteção para doenças crônicas por inquérito telefônico. Brasília, DF; 2017.

[32] Iser BPM, Vigo Á, Duncan BB, Schmidt MI. Trends in the prevalence of self-reported diabetes in Brazilian capital cities and the Federal District, 2006–2014. Diabetol Metab Syndr. BioMed Central; 2016;8: 70 10.1186/s13098-016-0185-x 27757172 PMC5064973

[33] Lackland Daniel T., et al Factors influencing the decline in stroke mortality: a statement from the American Heart Association/American Stroke Association. Stroke; 2014; 45 1: 315–353. 10.1161/01.str.0000437068.30550.cf 24309587 PMC5995123

[34] Kamal N, Lindsay MP, Côté R, Fang J, Kapral MK, Hill MD. Ten-year trends in stroke admissions and outcomes in Canada. Canadian Journal of Neurological Sciences. 2015 5;42(3):168–75. 10.1017/cjn.2015.20 25857318

[35] Mansur A de P, Favarato D. Mortality due to cardiovascular diseases in women and men in the five Brazilian regions, 1980–2012. Arq Bras Cardiol. SciELO Brasil; 2016;107: 137–146. 10.5935/abc.20160102 27437866 PMC5074067

