# Supplementary material for: Public hospitalizations for stroke in Brazil from 2009 to 2016
Source: PLoS One. 2019 Mar 19;14(3):e0213837. doi: 10.1371/journal.pone.0213837 (PMC6424448; doi:10.1371/journal.pone.0213837)
Supplement: S1 Table — (PDF) [file pone.0213837.s001.pdf]

S1 Table. **List of attributes from SIHSUS database used in this work.**

Attribute	Description
ano_cmpt	AIH processing year
mes_cmpt	AIH processing month
n_aih	AIH unique Id
Nasc	Date of birth of patient
Sexo	Gender of the patient (1 = male, 3 = female)
diag_princ	Principal diagnosis (ICD-10)
diag_secun	Secondary diagnosis (ICD-10)
diagsecN (1, 2, 3, 4, 5, 6, 7, 8)	List of secondary diagnoses (ICD-10)
cid_asso	Complementary causes diagnosis (ICD-10)
cid_morte	Cause of death diagnosis (ICD-10)
Cobranca	Discharge disposition reason (discharge to home, continue hospitalization, transfer, death etc.)
Morte	Indicates death of patient (0 = not died, 1 = died)
raca_cor	Race/Ethnicity of the patient (White = 01, Black = 02, Brown = 03, Yellow = 04, Indigenous = 05, No Information = 99)
dt_inter	Date of patient's hospitalization
ano_atendimento*	Year (4 digits) in which the patient was hospitalized - dt_inter yyyy
mês*	Month (2 digits) in which the patient was hospitalized - dt_inter mm
idade_real_anos*	Age of patient calculated by difference of date of admission (dt_inter) and date of birth (nasc)

Attributes with * are not original from SIHSUS, they were obtained from the original attributes in order to facilitate the analysis.
