# Supplementary material for: Public hospitalizations for stroke in Brazil from 2009 to 2016
Source: PLoS One. 2019 Mar 19;14(3):e0213837. doi: 10.1371/journal.pone.0213837 (PMC6424448; doi:10.1371/journal.pone.0213837)
Supplement: S2 Table — (PDF) [file pone.0213837.s002.pdf]

S2 Table. Population projection data obtained from the Brazilian Institute of Geography and Statistics for each year, age and sex.

MALE									FEMALE								
**AGE/YEAR**	2009	2010	2011	2012	2013	2014	2015	2016	**AGE/YEAR**	2009	2010	2011	2012	2013	2014	2015	2016
TOTAL	95776055	96706703	97610297	98487258	99336858	100159507	100955522	101726102	TOTAL	97767914	98791094	99786721	100755204	101695856	102609055	103495127	104355330
0	1599362	1576092	1553693	1532168	1511533	1491806	1472923	1454693	0	1527540	1504994	1483321	1462526	1442617	1423609	1405421	1387881
1	1617369	1593861	1570943	1548867	1527630	1507260	1487773	1469113	1	1546624	1523593	1501283	1479820	1459218	1439487	1420642	1402602
2	1639305	1615647	1592359	1569508	1547495	1526312	1505999	1486568	2	1568729	1545349	1522444	1500184	1478770	1458216	1438525	1419723
3	1661706	1638154	1614632	1591387	1568578	1546600	1525452	1505176	3	1591308	1567863	1544562	1521692	1499464	1478080	1457554	1437892
4	1683996	1660790	1637340	1613853	1590639	1567858	1545908	1524795	4	1613881	1590614	1567228	1543953	1521112	1498905	1477543	1457045
5	1705943	1683263	1660157	1636737	1613272	1590079	1567320	1545412	5	1636211	1613340	1590137	1566771	1543514	1520695	1498502	1477173
6	1727378	1705359	1682722	1659656	1636253	1612806	1589633	1566912	6	1658099	1635787	1612947	1589772	1566424	1543182	1520383	1498215
7	1748033	1726787	1704811	1682212	1659166	1635782	1612355	1589219	7	1679370	1657668	1635387	1612576	1589422	1566088	1542861	1520092
8	1768565	1747437	1726230	1704295	1681715	1658688	1635321	1611934	8	1700483	1678937	1657264	1635012	1612220	1589082	1565760	1542565
9	1750820	1767963	1746875	1725709	1703793	1681232	1658223	1634893	9	1687270	1700046	1678530	1656882	1634651	1611875	1588751	1565458
10	1754902	1750146	1767315	1746273	1725126	1703230	1680698	1657725	10	1690601	1686808	1699609	1678127	1656496	1634282	1611522	1588432
11	1753142	1754147	1749429	1766628	1745610	1724487	1702616	1680129	11	1690319	1690106	1686347	1699176	1677719	1656104	1633905	1611180
12	1750977	1752390	1753426	1748749	1765962	1744962	1723865	1702038	12	1689707	1689826	1689647	1685920	1698760	1677321	1655721	1633558
13	1749463	1750226	1751671	1752746	1748088	1765310	1744335	1723283	13	1689486	1689218	1689368	1689223	1685509	1698360	1676936	1655372
14	1748426	1748713	1749508	1750992	1752083	1747445	1764675	1743745	14	1689563	1688997	1688762	1688943	1688813	1685111	1697970	1676582
15	1746816	1746415	1746766	1747630	1749159	1750296	1745704	1762974	15	1689910	1688876	1688348	1688148	1688352	1688236	1684551	1697442
16	1745230	1743568	1743214	1743664	1744606	1746203	1747409	1742917	16	1691037	1689024	1688030	1687543	1687368	1687597	1687500	1683854
17	1744887	1741993	1740381	1740123	1740646	1741665	1743331	1744620	17	1693225	1690153	1688179	1687227	1686766	1686613	1686861	1686804
18	1745798	1741659	1738817	1737303	1737120	1737720	1738806	1740556	18	1696538	1692342	1689310	1687376	1686453	1686013	1685880	1686165
19	1745874	1742574	1738488	1735749	1734314	1734204	1734873	1736046	19	1698911	1695653	1691497	1688510	1686603	1685701	1685285	1685186
20	1742071	1741793	1738578	1734620	1731978	1730640	1730618	1731395	20	1698150	1697923	1694718	1690610	1687649	1685769	1684890	1684527
21	1745055	1737136	1736944	1733882	1730052	1727526	1726297	1726402	21	1705074	1697056	1696888	1693740	1689660	1686727	1684875	1684048
22	1759920	1740118	1732306	1732261	1729327	1725611	1723198	1722101	22	1724184	1703976	1696018	1695905	1692790	1688739	1685834	1684036
23	1780157	1754950	1735287	1727643	1727711	1724900	1721297	1719018	23	1749058	1723076	1702932	1695039	1694956	1691869	1687843	1684995
24	1798083	1775142	1750087	1730623	1723115	1723292	1720597	1717128	24	1770846	1747937	1722023	1701950	1694088	1694032	1690974	1687004
25	1817599	1792969	1770193	1745352	1726053	1718681	1718967	1716421	25	1793245	1769566	1746738	1720901	1700876	1693047	1693022	1690028
26	1817985	1812352	1787925	1765372	1740712	1721575	1714336	1714766	26	1797222	1791795	1768207	1745465	1719683	1699709	1691916	1691962
27	1789490	1812743	1807250	1783056	1760688	1736205	1717223	1710149	27	1773307	1795770	1790419	1766921	1744232	1718505	1698577	1690855
28	1741757	1784334	1807641	1802328	1778325	1756135	1731820	1713031	28	1730781	1771879	1794392	1789117	1765677	1743036	1717361	1697509
29	1695974	1736753	1779323	1802720	1797544	1773729	1751709	1727594	29	1689463	1729392	1770525	1793089	1787855	1764469	1741877	1716286
30	1648794	1690925	1731684	1774286	1797740	1792705	1769082	1747259	30	1646085	1687868	1727820	1768995	1791577	1786390	1763067	1740552
31	1602036	1643685	1685814	1726594	1769190	1792707	1787813	1764410	31	1603083	1644295	1686094	1726078	1767256	1789862	1784724	1761499
32	1559695	1597089	1638734	1680870	1721651	1764242	1787816	1783087	32	1564282	1601343	1642577	1684397	1724388	1765570	1788196	1783136
33	1520564	1554894	1592297	1633946	1676072	1716850	1759437	1783088	33	1528548	1562592	1599675	1640932	1682751	1722747	1763934	1786607
34	1479365	1515888	1550237	1587663	1629300	1671412	1712183	1754791	34	1490804	1526898	1560966	1598079	1639338	1681157	1721155	1762369
35	1436171	1474429	1510949	1545332	1582756	1624381	1666476	1707255	35	1451103	1488904	1525014	1559113	1596226	1637484	1679299	1719314
36	1399224	1430991	1469234	1505769	1540166	1577587	1619192	1661286	36	1417691	1448965	1486774	1522902	1557008	1594121	1635372	1677198
37	1372411	1394187	1425964	1464207	1500744	1535147	1572564	1614167	37	1394276	1415610	1446901	1484723	1520849	1554962	1592073	1633333
38	1352413	1367478	1389301	1421105	1459333	1495865	1530272	1567699	38	1377577	1392231	1413597	1444910	1482726	1518855	1552967	1590095
39	1332103	1347557	1362698	1384576	1416390	1454599	1491125	1525553	39	1360417	1375559	1390254	1411657	1442972	1480784	1516915	1551042
40	1311417	1326616	1342145	1357377	1379299	1411120	1449307	1485836	40	1342929	1357946	1373129	1387871	1409295	1440612	1478418	1514552
41	1291255	1305286	1320580	1336203	1351518	1373488	1405309	1443483	41	1326200	1340001	1355069	1370297	1385077	1406523	1437840	1475641
42	1271900	1285229	1299354	1314742	1330443	1345833	1367848	1399680	42	1310338	1323313	1337164	1352277	1367540	1382354	1403820	1435146
43	1252533	1265967	1279395	1293614	1309081	1324852	1340313	1362384	43	1294425	1307491	1320516	1334414	1349560	1364857	1379704	1401196
44	1233688	1246699	1260228	1273751	1288048	1303590	1319428	1334964	44	1278765	1291616	1304732	1317804	1331736	1346914	1362239	1377128
45	1214836	1226829	1239943	1253572	1267183	1281563	1297179	1313092	45	1263257	1275239	1288151	1301320	1314431	1328402	1343616	1358985

MALE									FEMALE								
**AGE/YEAR**	2009	2010	2011	2012	2013	2014	2015	2016	**AGE/YEAR**	2009	2010	2011	2012	2013	2014	2015	2016
46	1189131	1206964	1219071	1232293	1246015	1259717	1274181	1289877	46	1240935	1259017	1271065	1284043	1297258	1310413	1324427	1339686
47	1154877	1181430	1199340	1211558	1224873	1238684	1252471	1267016	47	1209516	1236781	1254903	1267014	1280040	1293298	1306496	1320560
48	1114897	1147408	1173974	1191956	1204272	1217676	1231562	1245436	48	1171940	1205464	1232745	1250911	1263072	1276141	1289440	1302688
49	1075868	1107693	1140171	1166757	1184793	1197205	1210687	1224652	49	1134958	1168016	1201534	1228829	1247023	1259232	1272338	1285690
50	1035272	1067408	1099197	1131638	1158226	1176327	1188840	1202414	50	1096571	1130277	1163314	1196814	1224106	1242330	1254590	1267744
51	993644	1025662	1057717	1089459	1121846	1148430	1166590	1179222	51	1057506	1091177	1124845	1157851	1191314	1218600	1236852	1249170
52	953749	984423	1016353	1048351	1080039	1112364	1138937	1157162	52	1019904	1052306	1085937	1119566	1152535	1185958	1213231	1231515
53	914956	944898	975491	1007361	1039291	1070918	1103177	1129738	53	983145	1014891	1047253	1080844	1114429	1147357	1180739	1208006
54	876465	906468	936327	966863	998662	1030521	1062076	1094276	54	946131	978314	1010021	1042344	1075886	1109426	1142312	1175656
55	837049	866692	896576	926349	956792	988490	1020243	1051704	55	908240	940419	972542	1004199	1036467	1069944	1103419	1136255
56	796410	826096	855598	885357	915007	945327	976890	1008512	56	869460	901707	933814	965861	997451	1029643	1063040	1096441
57	756379	785987	815522	844895	874519	904047	934238	965663	57	831046	863208	895372	927402	959373	990884	1023003	1056321
58	717049	746477	775924	805320	834554	864046	893446	923506	58	793079	825071	857144	889230	921173	953061	984499	1016541
59	679777	707658	736917	766214	795468	824557	853916	883186	59	756658	787373	819281	851261	883258	915115	946918	978283
60	643180	669181	696858	725907	755001	784057	812957	842129	60	720846	749921	780521	812307	844165	876045	907787	939488
61	604807	631482	657277	684713	713499	742338	771147	799809	61	683598	713148	742104	772561	804191	835894	867629	899236
62	565957	593804	620247	645817	673006	701530	730116	758678	62	645797	676295	705716	734537	764843	796316	827866	859459
63	527725	555657	583236	609430	634770	661712	689979	718311	63	608446	638899	669248	698522	727201	757356	788668	820070
64	492261	518121	545765	573062	599008	624119	650813	678826	64	573307	601939	632236	662426	691548	720085	750088	781245
65	456613	481280	506807	534081	561025	586662	611490	637882	65	537774	565519	593952	624036	654003	682932	711281	741090
66	424469	444465	468757	493875	520710	547240	572507	597003	66	506242	528871	556362	584541	614350	644053	672737	700860
67	399973	413175	432902	456799	481511	507916	534038	558941	67	482610	497855	520301	547543	575465	605002	634438	662884
68	380537	389331	402424	421859	445365	469679	495659	521382	68	464145	474614	489785	512048	539037	566702	595972	625147
69	362973	370410	379200	392157	411298	434421	458344	483910	69	447177	456458	466918	482011	504086	530825	558237	587245
70	345919	350984	358417	367146	379908	398671	421306	444737	70	430771	437670	446952	457388	472365	494191	520604	547688
71	325225	332174	337308	344687	353313	365825	384126	406179	71	410344	419533	426470	435737	446126	460952	482467	508483
72	301748	312303	319231	324388	331704	340220	352481	370333	72	386147	399642	408803	415775	425013	435346	450016	471226
73	277160	289764	300135	307005	312172	319412	327815	339828	73	360112	376086	389423	398551	405544	414748	425018	439528
74	255896	266155	278477	288644	295448	300609	307767	316048	74	337021	350735	366477	379663	388750	395754	404911	415116
75	234424	243246	253231	265180	275074	281776	286906	293947	75	313164	325731	339196	354653	367631	376649	383653	392740
76	212880	220435	228972	238607	250101	259669	266230	271308	76	289454	300240	312522	325700	340791	353517	362435	369426
77	194623	200180	207506	215758	225046	236102	245346	251756	77	269558	277516	288075	300094	312979	327715	340183	348998
78	178618	183014	188442	195538	203506	212455	223084	232015	78	252120	258448	266284	276631	288383	300981	315365	327580
79	164617	167963	172285	177575	184439	192127	200748	210965	79	236384	241734	247992	255712	265849	277338	289646	303686
80	150617	152640	155939	160142	165243	171806	179143	187358	80	219851	223698	228999	235174	242733	252594	263744	275681
81	134287	137507	139589	142802	146841	151705	157911	164834	81	199569	205136	209001	214243	220307	227659	237180	247916
82	117650	122595	125746	127827	130941	134816	139444	145310	82	178090	186218	191662	195544	200702	206635	213776	222961
83	101485	107404	112107	115151	117209	120218	123922	128324	83	156606	166175	173993	179318	183189	188251	194042	200969
84	88028	92646	98213	102656	105586	107610	110506	114044	84	138332	146130	155268	162796	167993	171829	176781	182427
85	75039	78701	82992	88121	92250	95029	96990	99739	85	119959	126458	133822	142443	149597	154618	158391	163193
86	62005	65516	68871	72771	77413	81181	83775	85642	86	101190	107213	113239	120106	128116	134825	139621	143296
87	51010	54136	57332	60385	63926	68125	71564	73974	87	85233	90441	96009	101636	108030	115469	121753	126319
88	41749	44537	47372	50269	53045	56254	60054	63190	88	71601	76184	80992	86171	91417	97368	104277	110157
89	34394	36453	38975	41536	44156	46679	49587	53026	89	60572	64001	68228	72697	77509	82396	87931	94349
90+	120175	126957	134638	143286	152780	163055	173888	185523	90+	252883	267130	282908	300584	320111	341555	364745	390047
