# Supplementary material for: Public hospitalizations for stroke in Brazil from 2009 to 2016
Source: PLoS One. 2019 Mar 19;14(3):e0213837. doi: 10.1371/journal.pone.0213837 (PMC6424448; doi:10.1371/journal.pone.0213837)
Supplement: S1 Appendix — (PDF) [file pone.0213837.s003.pdf]

S1 Appendix. Age-adjustment methodology.

For each year, we calculated the age-standardized estimates for total hospitalization, in-hospital mortality and case fatality rates using the age-adjustment methodology proposed by Ahmad et al. [1]. This procedure aims to standardize or age-adjust the in-hospital mortality and hospitalization rates and can be calculated as a weighted average of the age-specific rates, for each of the populations to be compared. The weights represent the relative age distribution of the standard external population. So, the age-adjustment methodology provides, for each population, a standardized rate that reflects the number of events that would have been expected if the populations being compared had had identical age distribution.

Therefore, the age-adjusted in-hospital mortality rate in 2009 and 2010, for example, can be calculated by the equations (1) and (2).

$$M^{2009} = \sum r_i^{2009} \left(\frac{n_i}{\sum_i n_i}\right) \quad (1)$$

$$M^{2010} = \sum r_i^{2010} \left(\frac{n_i}{\sum_i n_i}\right) \quad (2)$$

where n_i is the WHO World Standard population in the ith age group [1] (see Table below), r_i^{2009} and r_i^{2010} are the in-hospital mortality rates per 100,000 inhabitants for age group i in 2009 and 2010, respectively.

Regarding the age-adjusted hospitalization rate, the formulation is similar, as demonstrated in equations (3) and (4).

$$I^{2009} = \sum z_i^{2009} \left(\frac{n_i}{\sum_i n_i}\right) \quad (3)$$

$$I^{2010} = \sum z_i^{2010} \left(\frac{n_i}{\sum_i n_i}\right) \quad (4)$$

where z_i^{2009} and z_i^{2010} are the hospitalization rates per 100,000 inhabitants for age group i in 2009 and 2010, respectively.

In terms of case fatality, we divided the age-adjusted in-hospital mortality rate by the age-adjusted hospitalization rate, as demonstrated by the equations (5) and (6).

$$F^{2009} = \frac{M^{2009}}{I^{2009}} \quad (5)$$

$$F^{2010} = \frac{M^{2010}}{I^{2010}} \quad (6)$$

The Table below presents the WHO World Standard population for each age group (n_i) used in this work, as proposed by Ahmad et al. [1].

Table. Standard Population Distribution (percent). Adapted from Ahmad et al. [1].

Age Group	WHO World Standard (%)
0-4	8.86
5-9	8.69
10-14	8.60
15-19	8.47
20-24	8.22
25-29	7.93
30-34	7.61
35-39	7.15
40-44	6.59
45-49	6.04
50-54	5.37
55-59	4.55
60-64	3.72
65-69	2.96
70-74	2.21
75-79	1.52
80-84	0.91
85+	0.63
Total	100

Reference

[1] Ahmad OB, Boschi-Pinto C, Lopez AD, Murray CJ, Lozano R, Inoue M. Age Standardization of rates: A new who standard. In: Organization WH, editor. 2001. p. 1–14.
