# Supplementary material for: Public hospitalizations for stroke in Brazil from 2009 to 2016
Source: PLoS One. 2019 Mar 19;14(3):e0213837. doi: 10.1371/journal.pone.0213837 (PMC6424448; doi:10.1371/journal.pone.0213837)
Supplement: S3 Table — (PDF) [file pone.0213837.s004.pdf]

S3 Table. Formulation and description of the indicators used during the study.

Indicator	Formulation	Description
**Stroke admissions**	-	Number of hospital admissions with primary diagnosis of stroke[1]
**In-hospital deaths for stroke**	-	Number of stroke hospitalizations with outcome "death" in hospital
**Population**	-	Projection of the population for each year[2]
**Hospitalization rate[3]**	(stroke admission / Population) x 100,000	Number of stroke admissions per 100,000 inhabitants
**In-hospital mortality rate[3]**	(stroke deaths / Population) x 100,000	Number of stroke deaths per 100,000 inhabitants
**Case fatality rate[3]**	In-hospital mortality rate / Hospitalization rate	Proportion of stroke admissions with in-hospital death

[1] considering diagnosis codes from the International Statistical Classification of Diseases and Related Health Problems (ICD-10) categories I60, I61, I63 and I64. We considered only hospitalizations with patient outcomes of "discharge to home" or "death". Hospitalization registries with patients younger than 20 years old and with the length of stay (LOS) greater than 60 days were excluded.

[2] by IBGE, where population projections were calculated from the 2010 Demographic Census and information on birth and death records (see S2 Table);

[3]formulation without age-adjustment. The age-adjustment methodology is explained in details in the S1 Appendix.
